# Total Colonic Expulsion with Part of Small Bowel per Vaginum due to Low-Flow Phenomenon and NOMI: A Case Report

**DOI:** 10.1155/2011/498097

**Published:** 2011-04-03

**Authors:** Suha Deen, Martin Powell

**Affiliations:** ^1^Department of Histopathology, Level A, West Block, Queen's Medical Centre Campus, Nottingham University Hospitals, Derby Road, Nottingham NG7 2UH, UK; ^2^Department of Obstetrics and Gynaecology, Queen's Medical Centre, Nottingham, UK

## Abstract

*Introduction*. Intestinal ischaemia is a devastating disease process that could lead to bowel gangrene and death if either not diagnosed early or left untreated; death is usually caused by irreversible shock, intestinal necrosis, or septicaemia. It is usually seen in elderly patients with atherosclerotic disease. The course of bowel ischaemia may affect variable lengths of the intestine and it is not unusual for the condition to be followed by uneventful recovery. *Case presentation*. We are reporting an unusually rare case where an elderly patient passed an extraordinarily long segment of bowel, including rectum, the whole of the large bowel, and part of the small bowel, through anus following an episode of nonobstructive mesenteric ischaemia (NOMI) complicating myocardial infarction. To our knowledge, there are only eight cases reported in the literature where the condition was diagnosed upon the passage of short segments of the large bowel particularly of the rectosigmoid segment through the anus. *Conclusion*. Physician should keep a high level of suspicion in order to prevent it or at least recognise it early on and offer adequate management and hence reducing morbidity and mortality.

## 1. Case Report

An elderly female patient was treated for a fractured right neck of femur. Her Auston-Moore right hip replacement was uneventful. One week later, she became unwell with increasing shortness of breath and abdominal distension. A diagnosis of posterior myocardial infarction and pseudo-obstruction of the bowel was made and treated accordingly. Her general condition was fluctuating with recurrent diarrhoea, swinging temperature, and persistent confusion. Three weeks following her admission and while on the rehabilitation ward, a foul vaginal discharge was noted and a cordlike piece of tissue was seen protruding through the vagina. Later on, a piece of bowel was passed out through the vagina and placed in formalin pot following resection. The patient had had a vaginal shelf pessary fitted sometime ago. A diagnosis of vaginoenteric fistula was strongly suspected. The patient was too unwell for imaging studies such as MRI or CT scan. During this period and despite intensive treatment of her septicaemia, she continued to deteriorate and passed away six weeks following the hip replacement. A postmortem examination was refused by the relatives.

## 2. Histopathological Examination

### 2.1. Macroscopy

**Figure 1:**
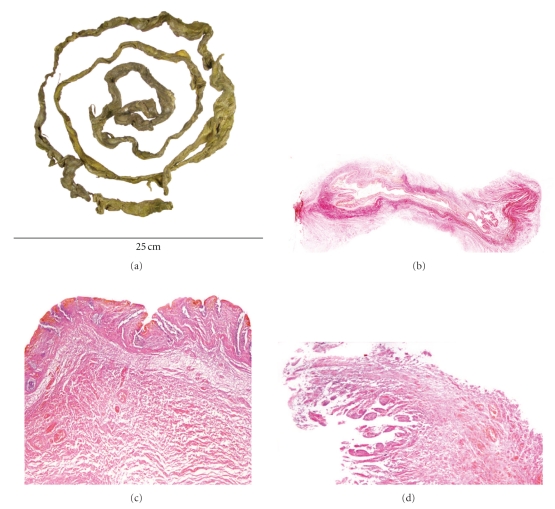
(a) Macroscopic view of the expelled loop of intestine as received showing pronounced degenerative and ischaemic changes. (b) Low power view of a section of the infarcted large bowel; EVG special stain. (c) High power view of (b) showing oedematous and necrotic tissue. (d) One end of the specimen showing small bowel mucosa.

The specimen was a long cordlike greenish yellow tissue measuring 1350 × 20 mm maximum without any identifiable viable tissue (Figure 1(a)). 

### 2.2. Microscopic Examination

Microscopic examination of sections of the tissue revealed predominantly large and partly small bowel tissue with different and variable thickness ranging from mucosa and submucosa up to and including the inner and outer layers of the muscularis propria (Figures 1(b), 1(c), and 1(d)).

## 3. Discussion

Spontaneous passage of a large bowel cast caused by acute ischaemic injury is an extraordinary complication of mesenteric ischaemia. A literature search through PubMed using the keywords colon, rectum, ischaemia, infarction, sloughed, passed, per vaginam, and per rectum revealed 8 cases in which a short segment ranging between 25 cm of recto-sigmoid and 96 cm^3^ of descending colon down to the upper rectum was passed per anum [1–6]. However, in our interesting case not only was there a spontaneous expulsion of the whole of the colon and part of the small bowel to a length of 135 cm but also the bowel cast expelled per vaginum. 

Of the eight reported cases, ischaemic complication occurred following abdominal aortic aneurysm repair in five cases; all patients survived this complication except for one case of a 67-year-old male who passed a full thickness cast of sigmoid colon 25 cm long following abdominal aortic aneurysm repair; the latter case was reported by Sado et al. in J Jpn Soc Colorectal Dis (in Japanese) [1]. Whilst in seven of these cases, as well as our case, infarcted muscularis propria was also found in addition to necrotic mucosa and submucosa [1–6].

 The mortality following bowel ischaemia remains high although it has dropped from 85% to 60% over the last 30 years due to advances in early diagnosis and management. However, the latter, as expected, has led to increase in the incidence of this entity [7]. 

The aetiology of bowel ischaemia is obviously secondary to a compromise in blood flow to the bowel. This may be attributed to a predisposing obstructive factor causing segmental ischaemia or has a non-obstructive aetiology. The latter is usually secondary to cardiovascular events and could affect the major supply of the celiac axis and superior and inferior mesenteric arteries. The likely causes of non-obstructive intestinal ischaemia include

1. dissecting aortic aneurysm with secondary compression of both main mesenteric arteries,
2. vasospasm of both mesenteric arteries secondary to medications for example, digoxin, ergot, and cocain intoxication [7],
3. low-flow phenomenon precipitated by hypovolaemia, severe hypotension, or shock [8],
4. haematological clotting causes predisposing to excessive clotting mechanism.

In our case report, there was no history of clotting disorder, vasoactive medication, or aortic dissection. Other than the unlikely event of two simultaneous and separate thrombi of the superior and inferior mesenteric arteries, it seems that low-flow phenomenon complicating myocardial infarction and progressing to extensive intestinal tissue necrosis and pronounced sloughing of this tremendous length of bowel is the most likely pathological explanation to what had happened to our patient. Mortality is usually very high if this unexpected extensive and severe intestinal infarction presented together with the recent myocardial infarction.

NOMI accounts for 15–20% of acute cases of intestinal ischaemia and is mostly seen in patients who are already critically ill [7]. Systemic hypotension is often followed by splanchnic and peripheral vasoconstriction. This physiological response will predispose to NOMI particularly in the elderly population whose vascular bed is already compromised by systemic atherosclerosis [9]. 

This lady had had a vaginal ring pessary fitted for uterine prolapse. The clinical history is clear that there was a significant foul vaginal discharge and a cordlike pieces of tissue protruding from the vagina. It is clinically recognised that neglected vaginal pessary in certain circumstances might erode through vaginal wall predisposing to a fistula [10].

## 4. Conclusion

This is a very unusual case of extensive non-obstructive intestinal ischaemia affecting the area supplied by the superior and inferior mesenteric arteries, that is, including rectum, the whole of the large bowel, and part of the small bowel. Ischaemic change was most likely secondary to a low flow phenomenon precipitated by prior myocardial infarction. Physician should keep a high level of suspicion in order to prevent it or at least recognise it early on and offer adequate management and hence reducing morbidity and mortality.
